# Persistent Rhinosinusitis-Like Symptoms Revealing a Lactotroph Pituitary Neuroendocrine Tumour: A Case Report

**DOI:** 10.7759/cureus.112575

**Published:** 2026-07-13

**Authors:** Christopher Chang, Kristy S Samlal, Nalini D Maharaj, Ananda Hanooman, Sriram Nallamothu

**Affiliations:** 1 Department of Primary Health Care, South-West Regional Health Authority, San Fernando, TTO; 2 Department of Primary Health Care, South-West Regional Health Authority, Siparia, TTO; 3 Department of Primary Health Care, South-West Regional Health Authority, Penal, TTO; 4 Department of Radiology, South-West Regional Health Authority, San Fernando, TTO; 5 Department of Internal Medicine, South-West Regional Health Authority, Point Fortin, TTO

**Keywords:** chronic cough, chronic rhinosinusitis, incidental sellar mass, lactotroph pitnet, pituitary macroadenoma, pituitary neuroendocrine tumour (pitnet), postnasal drip, primary care, upper airway cough syndrome

## Abstract

Pituitary neuroendocrine tumours (PitNETs) commonly present with symptoms related to mass effect or hormonal dysfunction, including headache, visual disturbances, and endocrine abnormalities. However, atypical presentations without neurological or hormonal “red flag” symptoms may delay diagnosis, particularly in primary care settings where benign upper respiratory conditions are more frequently encountered.

We report the case of a 56-year-old postmenopausal woman who presented with a five-month history of persistent postnasal drip, chronic non-productive cough and a sensation of nasal congestion characterised by fullness in the nasal and retro-orbital region. Despite conservative medical management, persistent symptoms prompted a computed tomography (CT) of the brain and paranasal sinuses, which unexpectedly revealed a sellar mass extending into the left sphenoid sinus. Subsequent magnetic resonance imaging (MRI) confirmed a 3.4cm pituitary macroadenoma with left cavernous and sphenoid sinus invasion, left internal carotid artery encasement, optic nerve involvement, and imaging features of pituitary apoplexy. Hormonal evaluation showed hyperprolactinaemia, and histopathology following endoscopic endonasal resection confirmed a lactotroph PitNET.

This case highlights the importance of diagnostic reassessment in patients with persistent symptoms and demonstrates that clinically significant PitNETs can masquerade behind common benign upper respiratory conditions.

## Introduction

Pituitary neuroendocrine tumours (PitNETs), previously referred to as pituitary adenomas, are among the most common intracranial neoplasms. They account for approximately 10%-15% of intracranial tumours and typically arise in the sella turcica in the anterior pituitary lobe, typically occurring in females between 40 and 70 years [1,2]. The prevalence of clinically significant pituitary adenomas is estimated to be approximately 89 per 100,000 individuals, with prolactin-secreting tumours representing the most common subtype in approximately 50% [3]. Despite their relative frequency, diagnosis is often delayed because symptoms may develop insidiously and remain nonspecific for prolonged periods. Incidental tumours are found in nearly 14.4% of autopsies and 22.5% of radiological studies [2].

Typically, pituitary macroadenomas present with symptoms related to mass effect or hormonal dysfunction. Common neurological manifestations include headaches, visual disturbances, cranial nerve palsies, and visual field defects, particularly bitemporal hemianopia resulting from optic chiasm compression. Visual impairment has been reported in approximately 40%-60% of patients with pituitary macroadenomas, while headache remains one of the most frequently reported presenting complaints in 15% of patients [4,5].

In contrast, atypical presentations without overt neurological or endocrine “red flag” symptoms may obscure the diagnosis and contribute to delayed recognition. This diagnostic dilemma is particularly relevant in primary care and low-resource settings, where access to advanced imaging and specialist evaluation may be limited, and common benign conditions are encountered far more frequently than intracranial pathology. Symptoms such as chronic cough, postnasal drip, nasal congestion, and presumed chronic rhinosinusitis are routinely managed conservatively, often appropriately, but may occasionally conceal more significant underlying pathology.

We present a case of a 56-year-old woman who originally presented to a primary care facility with chronic upper airway symptoms suggestive of upper airway cough syndrome secondary to chronic rhinosinusitis. Absence of visual symptoms, focal neurological deficits, or overt endocrine abnormalities initially masked the presence of a pituitary macroadenoma. This case highlights the importance of maintaining clinical vigilance when evaluating persistent or refractory symptoms, even when the preliminary presentation appears benign. It underscores the diagnostic challenges faced in resource-constrained primary care environments.

## Case presentation

A 56-year-old postmenopausal woman with a medical history of hypertension, dyslipidaemia, chronic venous insufficiency, and prediabetes presented to a primary care facility with a five-month history of persistent postnasal drip, chronic non-productive cough and a sensation of nasal congestion characterised by fullness in the nasal and retro-orbital region. These symptoms had progressively worsened and adversely affected her quality of life. She also reported frequent throat clearing but denied purulent nasal discharge, epistaxis, anosmia, or facial swelling. There were no associated constitutional symptoms, including fever, headaches, unintentional weight loss, or night sweats. She further denied wheeze, dyspnoea, gastro-oesophageal reflux symptoms, and visual disturbances. The remainder of the history was unremarkable.

On examination, the patient was haemodynamically stable. The vital signs were within normal limits with a blood pressure of 137/92 mmHg, a pulse rate of 74 beats per minute, a respiratory rate of 18 breaths per min, an oxygen saturation of 99% on room air and a random blood glucose level of 93 mg/dL. Head and neck examination revealed mild maxillary sinus tenderness with preserved facial symmetry. No nasal polyps, significant septal deviation, or cervical lymphadenopathy were identified. The oropharynx was mildly erythematous. Respiratory examination was unremarkable, with equal air entry bilaterally and no adventitious breath sounds. Neurological examination was unremarkable, with no focal deficits or visual abnormalities detected at that time.

Initial laboratory investigations were unremarkable, with normal haematological parameters, renal function, serum electrolytes, and liver function tests. Lipid profile findings were consistent with hypercholesterolaemia, demonstrating elevated total cholesterol as well as low-density lipoprotein levels. Glycated haemoglobin findings were in keeping with prediabetes (Table 1). A chest radiograph was also performed and showed no significant abnormalities.

**Table 1:** Laboratory parameters on initial presentation BUN: blood urea nitrogen; AST: aspartate aminotransferase; ALT: alanine aminotransferase; LDL: low-density lipoprotein; HDL: high-density lipoprotein; HbA1c: glycated haemoglobin

Blood Parameters	Patient Values	Reference Range
Haemoglobin	14.3	11.7 – 15.5 g/dL
White Blood Cells	5.83	4.1 – 11.2 x 10^3 ^µL
Platelets	216	159 – 388 x 10^3 ^µL
Sodium	143	135.0 – 145.0 mmol/L
Potassium	4.9	3.5 – 5.1 mmol/L
Chloride	103.7	97.0 – 110.0 mmol/L
Creatinine	0.7	0.5 – 0.9 mg/dL
BUN	14	6 – 23 mg/dL
AST	22	5.0 – 40.0 IU/L
ALT	33.6	5.0 – 41.0 IU/L
Albumin	4.26	3.50 – 5.20 g/dL
Cholesterol	223.5	170.00 – 200.00 mg/dL
Triglycerides	108.7	40.00 – 150.00 mg/dL
LDL	152.56	40.00 – 130.00 mg/dL
HDL	49.2	45.00 – 65.00 mg/dL
HbA1c	6.2	4.8 – 5.9 %

An initial clinical diagnosis of upper airway cough syndrome secondary to chronic rhinosinusitis was made, with differentials including cough-variant asthma and upper airway allergy. Conservative management was commenced with daily nasal saline irrigation, intranasal budesonide 64 mcg, one spray per nostril daily, and loratadine 10 mg once daily. A two-week follow-up was scheduled.

At the follow-up review, the patient reported persistent symptoms with minimal clinical improvement despite adherence to therapy. Given the chronic and refractory nature of her presentation, further evaluation was conducted. A non-contrast CT brain and paranasal sinuses was requested, and the patient was referred to the Ear, Nose, and Throat outpatient clinic for specialist assessment.

**Figure 1:**
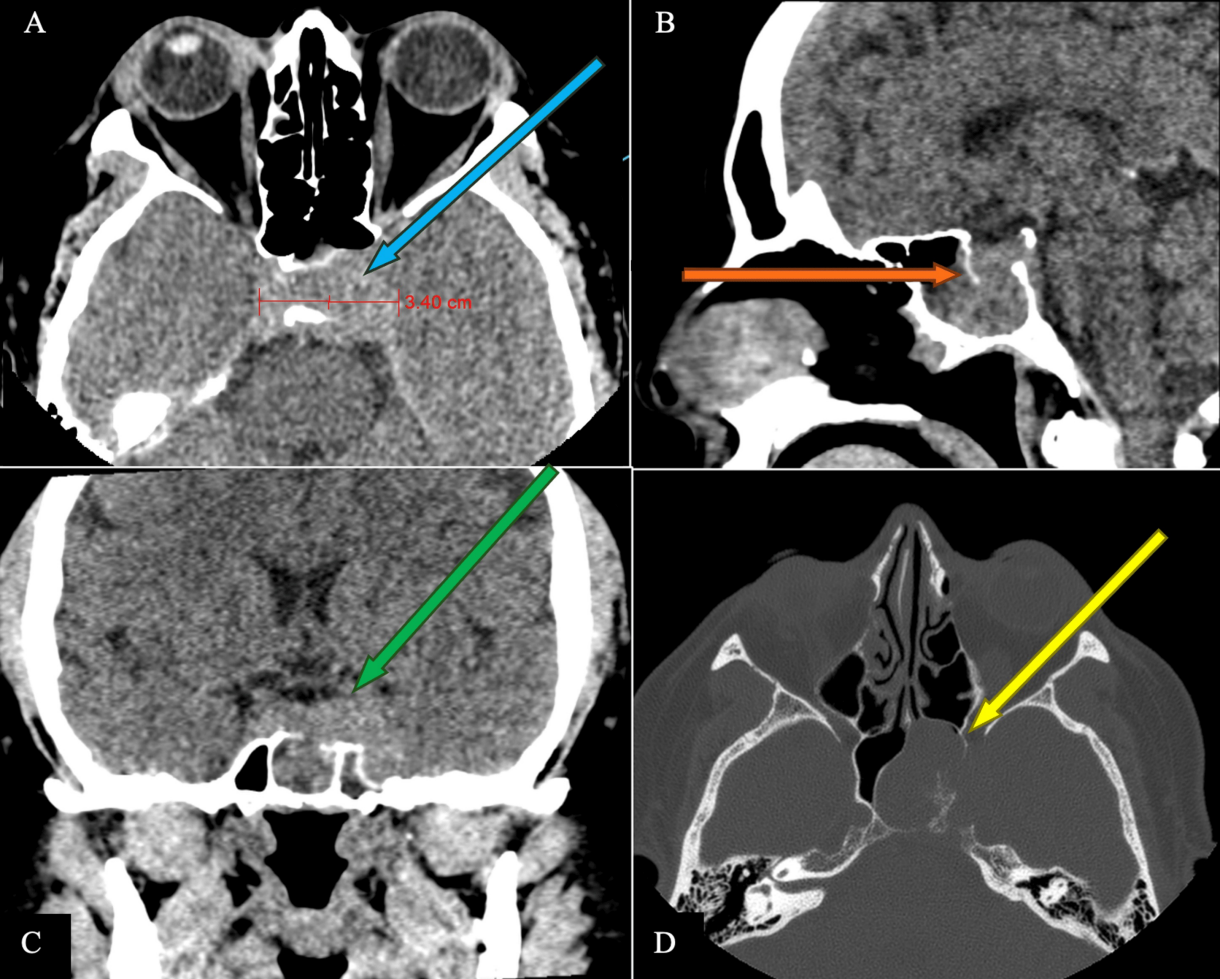
(A) Axial CT of the brain and paranasal sinuses shows a mildly hyperattenuating, soft tissue, expansile mass arising from the sella turcica (blue arrow). (B) Sagittal CT image demonstrates expansion through the floor of the sella turcica (orange arrow) with extension into the left sphenoid sinus inferiorly. (C) Coronal CT image shows lateral extension into the left middle cranial fossa (green arrow). (D) Axial bone window CT image shows expansile remodelling of the sphenoid bone (yellow arrow). CT: computed tomography

Non-contrast CT of the brain and paranasal sinuses demonstrated an expansile mass arising from the sella turcica measuring 3.4 cm (transverse (TS)) × 3.2 cm (anteroposterior (AP)) × 2.3 cm (craniocaudal (CC)). The lesion extended through the sellar floor into the left sphenoid sinus and laterally toward the left middle cranial fossa, with associated remodelling of the adjacent sphenoid bone (Figure 1). These findings were suspicious for a pituitary macroadenoma, requiring an MRI of the brain with pituitary protocol for further characterisation.

**Figure 2:**
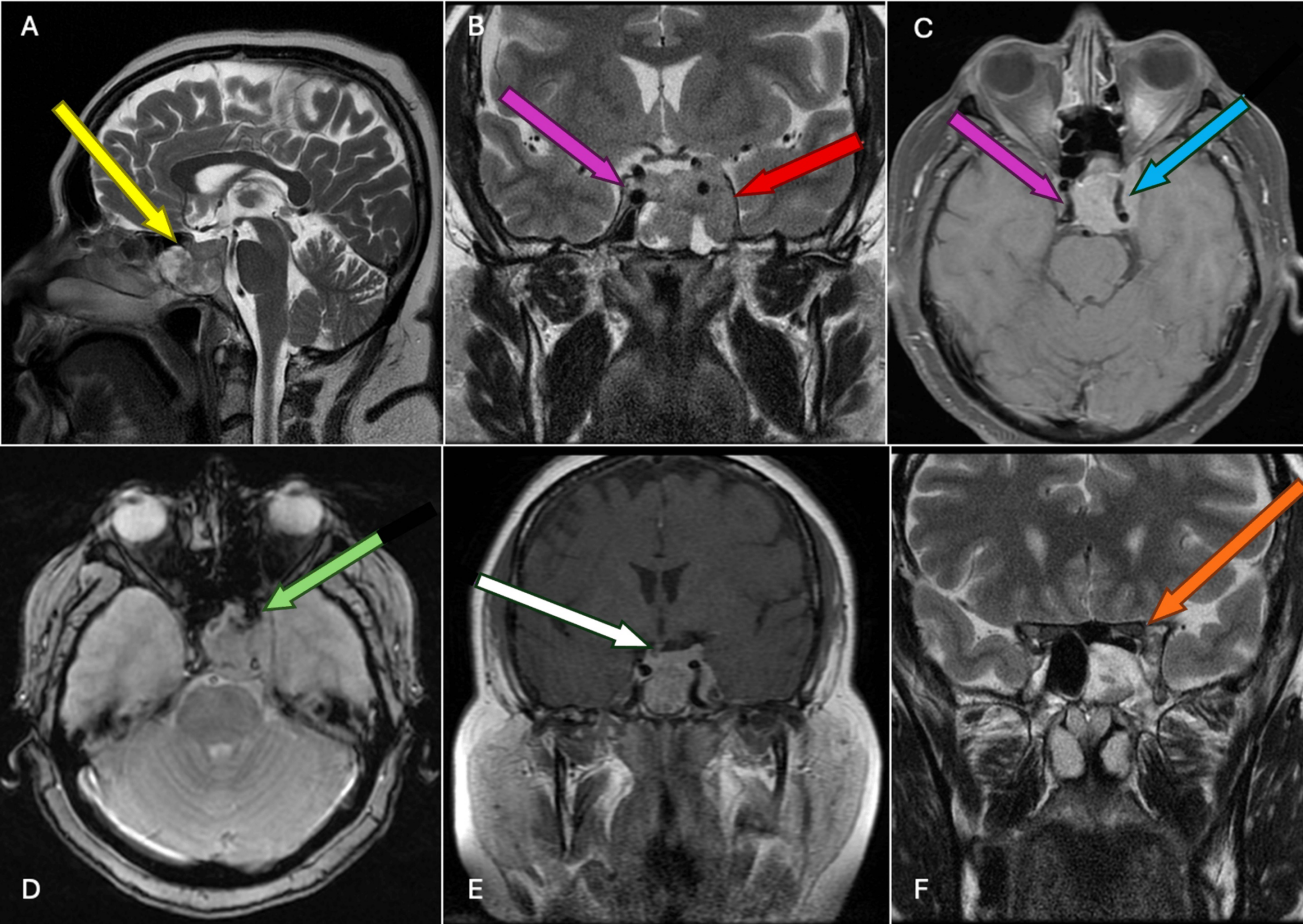
(A) Sagittal T2-weighted MRI of the brain demonstrates a well-defined, heterogeneous lesion occupying the sella turcica with inferior extension into the left sphenoid sinus (yellow arrow). (B) Coronal T2-weighted image shows extension into the left cavernous sinus with early superior extension into the middle cranial fossa (red arrow) and medial abutment of the right ICA (purple arrow). (C) Axial T1 post-contrast image demonstrates encasement of the left ICA (blue arrow) and medial abutment of the right ICA (purple arrow). (D) Axial T2-weighted GRE image demonstrates susceptibility artefact within the lesion, in keeping with haemorrhagic components suggestive of pituitary apoplexy (green arrow). (E) Coronal T1 post-contrast image demonstrates rightward deviation of the pituitary stalk (white arrow), which is continuous with the lesion and undifferentiable. (F) Coronal T2-weighted image demonstrates encasement of the ipsilateral optic nerve (orange arrow). MRI: magnetic resonance imaging; GRE: gradient recalled echo; ICA: internal carotid artery

The patient subsequently sought further evaluation abroad, where the brain MRI with pituitary protocol demonstrated a well-defined, heterogeneous lesion measuring 3.4cm (TS) × 3.2cm (AP) × 2.5cm (CC) within the sella turcica. The lesion extended inferiorly into the left sphenoid sinus and laterally to the left cavernous sinus with encasement of the left internal carotid artery and ipsilateral optic nerve. Early extension into the left middle cranial fossa was also identified. The lesion showed heterogeneous post-contrast enhancement with internal haemorrhagic components and mild diffusion restriction, consistent with pituitary apoplexy (Figure 2).

Hormonal evaluation demonstrated elevated serum prolactin levels, while other measured anterior pituitary hormonal parameters were within their respective reference ranges (Table 2). These findings supported the initial working diagnosis of a lactotroph PitNET.

**Table 2:** Baseline hormonal assessment of pituitary function T4: thyroxine; TSH: thyroid-stimulating hormone; ACTH: adrenocorticotropic hormone; IGF-1: insulin-like growth factor 1; FSH: follicle-stimulating hormone; LH: luteinizing hormone

Blood Parameters	Patient Values	Reference Range
Total T4	6.7	5.1 – 14.1 µg/dL
TSH	1.15	0.27 – 4.20 µIU/mL
ACTH	16.7	7.2 – 63.3 pg/mL
Cortisol (9AM)	9.7	6.02 – 18.40 µg/dL
Growth Hormone	0.17	<9.88 ng/mL
IGF-1	103.8	67.3 – 201.0 ng/mL
Prolactin	34.3	4.8 – 23.3 ng/mL
FSH	61.2	Post menopause: 25.8 – 134.8 mIU/mL
LH	31	Post menopause: 7.7 – 58.8 mIU/mL

**Figure 3:**
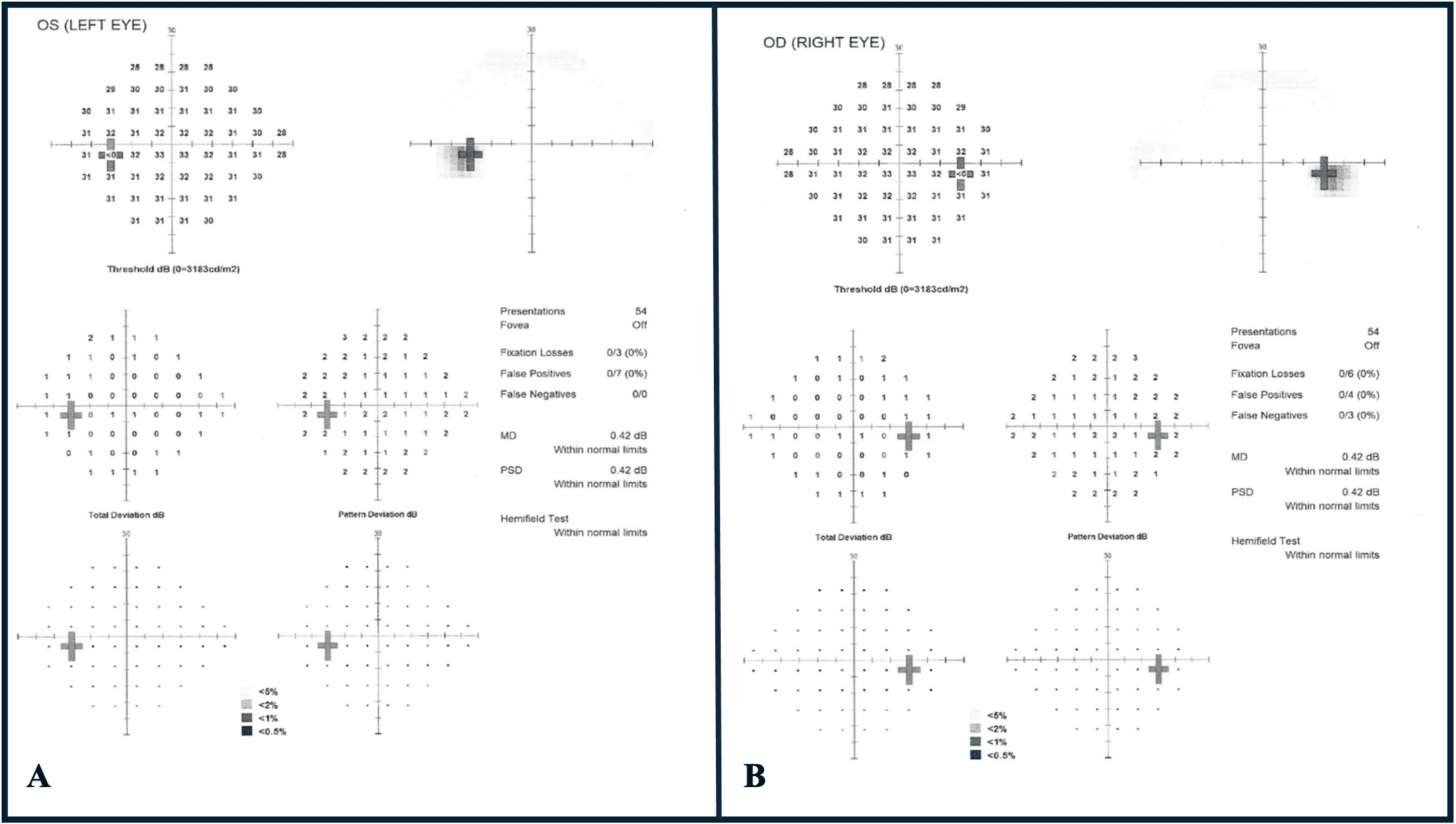
Formal automated perimetry (Henson 9000, 30/24-2 threshold program) demonstrates normal visual fields bilaterally, with mean deviation, pattern standard deviation and hemifield analyses all within normal limits. (A) Left eye. (B) Right eye. OS: left eye; OD: right eye; MD: mean deviation; PSD: pattern standard deviation

Formal automated perimetry was performed and demonstrated normal visual fields bilaterally, with mean deviation, pattern standard deviation, and hemifield analyses all within normal limits (Figure 3).

Treatment

The patient underwent endoscopic endonasal resection of the tumour. Histopathological examination confirmed a PitNET, consistent with a lactotroph subtype. Immunohistochemistry supported this diagnosis with diffuse prolactin and PIT-1 positivity, in keeping with lineage differentiation. The Ki-67 proliferation index was estimated at 3%, indicating a low-to-intermediate proliferative activity. Postoperatively, serum prolactin levels demonstrated a decline from 23.7 ng/mL on day one to 4.5 ng/mL on day two. Cabergoline therapy was initiated at 0.25mg twice weekly orally.

Outcome and follow-up

At approximately six months postoperatively, during routine follow-up at the Chronic Disease Clinic, the patient reported a significant reduction in the severity of her presenting symptoms, including a decrease in postnasal drip, non-productive cough, and the sensation of nasal and retro-orbital fullness. Although her symptoms had not completely resolved, she described feeling substantially better, with an overall improvement in her quality of life. She continues to undergo follow-up in both Neurosurgery and Endocrinology outpatient clinics. She is awaiting repeat brain MRI, hormonal evaluation, and serial prolactin monitoring to assess for residual or recurrent disease and ongoing treatment response.

## Discussion

PitNETs are often diagnostically challenging due to their broad clinical manifestations ranging from incidental radiological findings to significant neurological and endocrine dysfunction, largely reflecting their indolent progression [6]. Although pituitary macroadenomas traditionally present with symptoms related to compression of adjacent structures or abnormal hormonal secretion, many are asymptomatic until they reach advanced stages. Delayed diagnosis can lead to tumour growth and local invasion, requiring aggressive treatment, reduced treatment success and poorer prognosis [7]. This case illustrates that significant intracranial pathology may remain clinically silent, with atypical manifestations masquerading as a common benign upper respiratory condition, thereby creating a diagnostic challenge in primary care settings where such respiratory conditions are far more prevalent.

In this case, the patient's first presentation of persistent postnasal drip, non-productive cough and a sensation of fullness involving the nasal and retro-orbital regions was highly suggestive of upper airway cough syndrome secondary to chronic rhinosinusitis or allergic rhinitis, both of which are among the leading causes of chronic cough in ambulatory practice [8]. Importantly, she did not report any headaches, visual disturbances, galactorrhoea, menstrual abnormalities, or focal neurological deficits that would typically prompt consideration of a pituitary pathology. Other common differential diagnoses included cough-variant asthma, gastro-oesophageal reflux disease, and chronic respiratory infection, establishing that the preliminary conservative management was appropriate and consistent with current primary care recommendations [8].

Despite radiological evidence of a tumour measuring more than 3 cm with left sphenoid and cavernous sinus invasion, internal carotid artery encasement, optic nerve involvement, and early middle cranial fossa extension, the patient’s neurological function was preserved with no visual complaints or objective visual field defects on formal automated perimetry. This discordance between tumour size, radiological invasiveness, and clinical presentation has been described previously, with some pituitary macroadenomas remaining clinically silent until identified incidentally during imaging performed for unrelated indications [7, 9]. The present case further demonstrates that the absence of neurological or endocrine "red flag" symptoms does not exclude extensive local disease.

One of the most notable features of this case was the tumour's inferior extension through the sellar floor into the sphenoid sinus. PitNETs typically enlarge superiorly toward the optic chiasm, whereas inferior extension into the sphenoid sinus represents a relatively uncommon growth pattern [10]. Involvement of the sphenoid sinus may produce non-specific sinonasal symptoms, including nasal congestion, postnasal drip, facial pressure, rhinorrhoea, recurrent sinusitis, epistaxis and chronic cough, closely resembling benign upper respiratory conditions as seen in this case [11]. Consequently, patients may initially present to primary care physicians or otolaryngologists rather than specialists in endocrinology, neurology, or neurosurgery, potentially delaying recognition of the underlying intracranial pathology.

The modest elevation in serum prolactin despite the relatively large tumour size represents a diagnostic consideration. Possible explanations include pituitary stalk effect secondary to tumour compression, partial secretory activity of a lactotroph PitNET, or an assay-related phenomenon such as the high-dose hook effect, which was not excluded by serial dilution in this case [12]. However, histopathological confirmation of diffuse prolactin and PIT-1 expression established the diagnosis of a lactotroph PitNET. Notably, serum prolactin levels decreased with postsurgical resection, supporting the functional nature of the lesion and indicating effective early tumour decompression. This rapid biochemical response is consistent with reports demonstrating postoperative prolactin normalisation within 72 hours following resection of prolactin-secreting tumours and relief of stalk compression [12].

Although dopamine receptor agonists, particularly cabergoline, are the first-line treatment for most lactotroph PitNETs, management should be individualised according to tumour size, invasiveness, and clinical presentation [6, 13]. In this case, the combination of a large invasive sellar mass, disproportionate mild hyperprolactinaemia, extensive skull base involvement, and radiological features suggestive of pituitary apoplexy prompted surgical resection for tumour decompression and definitive histological diagnosis. Cabergoline was subsequently initiated following confirmation of lactotroph PitNET.

From a primary care perspective, this case highlights several important clinical lessons. While common conditions should remain the most likely diagnoses, persistence of symptoms despite guideline-directed therapy should prompt reassessment of the working diagnosis. Chronic cough and presumed chronic rhinosinusitis that fail to respond to appropriate medical management warrant further investigation, particularly when symptoms are prolonged or progressively impair quality of life. Although advanced imaging is not routinely indicated in uncomplicated sinonasal disease, clinicians should maintain a low threshold for further evaluation in refractory or atypical presentations to exclude less common structural pathology.

The literature describing PitNETs presenting predominantly as chronic upper airway cough syndrome or chronic rhinosinusitis remains limited. Most published reports involve presentations with visual impairment, pituitary apoplexy, cranial neuropathies, endocrine dysfunction, or incidental radiological discoveries [4,5,9]. In contrast, the patient presented with a common primary care complaint, delaying suspicion of intracranial pathology until advanced imaging was obtained. By highlighting an unusual clinical presentation associated with inferior sphenoid sinus extension, this case expands the spectrum of recognised PitNET presentations and reinforces the importance of considering alternative diagnoses when common conditions fail to follow their expected clinical course. This case has several limitations. Firstly, although the patient demonstrated mild hyperprolactinaemia despite a large invasive sellar mass, serial dilution of the prolactin assay was not performed to exclude the possibility of a high-dose hook effect, an important laboratory phenomenon that may result in falsely low prolactin measurements in patients with markedly elevated hormone concentrations. This represents an important consideration when interpreting discordant biochemical and radiological findings in suspected macroprolactinomas. Secondly, the original histopathological and immunohistochemical images were unavailable for inclusion, as only the final pathology report was accessible from the treating institution. Therefore, the diagnosis of lactotroph PitNET was based on the documented histopathological findings and immunohistochemical results demonstrating prolactin and PIT-1 positivity.

Ultimately, this case reinforces an important principle in primary care: while rare diagnoses are not expected at initial presentation, persistence of symptoms despite appropriate management should prompt reconsideration of the working diagnosis. Early recognition of uncommon but clinically significant pathology, such as pituitary macroadenomas, may prevent further tumour progression and improve patient outcomes.

## Conclusions

This case demonstrates that persistent rhinosinusitis-like symptoms in a primary care setting ultimately led to the diagnosis of a PitNET. Although PitNETs typically present with neurological, visual, or endocrine manifestations, they may remain clinically silent despite extensive local invasion. The absence of classical "red flag" features in this patient contributed to an initial conservative management approach and delayed intracranial imaging. This case underscores the importance of diagnostic reassessment when common symptoms persist despite appropriate treatment and highlights the value of maintaining a broad differential diagnosis to facilitate the timely recognition and avoid delayed diagnosis of significant intracranial pathology.
